# The emerging role of RNA N6-methyladenosine methylation in breast cancer

**DOI:** 10.1186/s40364-021-00295-8

**Published:** 2021-05-27

**Authors:** Fangchao Zheng, Feng Du, Jiuda Zhao, Xue Wang, Yiran Si, Peng Jin, Haili Qian, Binghe Xu, Peng Yuan

**Affiliations:** 1 Department of Medical Oncology, National Cancer Centre/National Clinical Research Center for Cancer/Cancer Hospital, Chinese Academy of Medical Sciences and Peking Union Medical College, No. 17 Panjiayuan Nanli, Beijing, 100021 China; 2 Key Laboratory of Carcinogenesis and Translational Research (Ministry of Education/Beijing), The VIPII Gastrointestinal Cancer Division of Medical Department, Peking University Cancer Hospital and Institute, Beijing, 100021 China; 3 Breast Disease Diagnosis and Treatment Center, Affiliated Hospital of Qinghai University & Affiliated Cancer Hospital of Qinghai University, Xining, 810000 China; 4 Department of VIP Medical Services, National Cancer Centre/National Clinical Research Center for Cancer/Cancer Hospital, Chinese Academy of Medical Sciences and Peking Union Medical College, Beijing, 100021 China; 5 Department of Surgery, National Cancer Centre/National Clinical Research Center for Cancer/Cancer Hospital, Chinese Academy of Medical Sciences and Peking Union Medical College, Beijing, 100021 China; 6 State Key Laboratory of Molecular Oncology, Cancer Hospital/Institute, Chinese Academy of Medical Sciences and Peking Union Medical College, Beijing, 100021 China

**Keywords:** RNA methylation, Breast cancer, Clinicopathological features, Prognosis, Drug target

## Abstract

N6-methyladenosine (m6A) modification is the most prevalent internal mRNA modification and is involved in many biological processes in eukaryotes. Accumulating evidence has demonstrated that m6A may play either a promoting or suppressing role in breast cancer, including in tumorigenesis, metastasis and angiogenesis. In this review, we summarize the latest research progress on the biological function and prognostic value of m6A modification in breast cancer, as well as potential related therapeutic strategies.

## Highlights

1. N6-methyladenosine (m6A) modification can promote or suppress cell growth in breast cancer tissues and cells.
2. m6A modification is associated with the clinicopathological features and prognosis of breast cancer.
3. m6A modification is involved in drug efficacy and may be a potential selective therapeutic target in breast cancer.

## Background

According to GLOBOCAN 2018, breast cancer is the most commonly diagnosed cancer and the leading cause of cancer death in females [1]. Recently, novel types of drugs have provided effective treatment strategies in the prevention and control of breast cancer, including immune checkpoint inhibitors for PD-1/PD-L1/TMB/CTLA4, PARP inhibitors for BRCA mutation and HRD, CDK4/6 inhibitors against CDK4/6, PI3Kα inhibitors and AKT inhibitors targeting the PI3K/AKT/PTEN pathway, and antibody-drug conjugates targeting HER2 [2–6]. In addition, great progress has been made in whole-exome sequencing, miRNA sequencing, and single nucleotide polymorphism, DNA methylation, and reverse-phase protein array analyses in recent years, which provide some new methods for the diagnosis and treatment of breast cancer [7–10]. However, global cancer statistics still demonstrate that there were 2,088,849 new cases and 626,679 cancer deaths worldwide in 2018 [1]. In addition, WHO CANCER TOMORROW predicted that approximately 817,361 females could die from breast cancer by 2030. That is, the prognosis of breast cancer remains concerning. Therefore, there is still an urgent need for effective therapies or regimens.

To enhance the efficacy of breast cancer treatment, it is necessary to understand the occurrence, development and molecular biological characteristics of breast cancer. In recent years, many studies have been conducted, but additional research is still required to determine the potential mechanism of oncogenesis and progression of breast cancer. RNA modification, especially N6-methyladenosine (m6A) modification, has provided a more effective method and new prospects in the treatment of breast cancer [11].

Emerging evidence suggests that m6A modification is associated with tumour proliferation, differentiation, tumorigenesis, invasion and metastasis and functions as either an oncogene or anti-oncogene in breast cancer [12–15]. Despite its functional significance in tumour generation processes, the critical roles of m6A modifications in breast cancer remains uncertain. This article briefly reviews the mechanism, potential therapeutic strategies and possible prognostic implications of m6A modification in breast cancer.

### The molecular mechanisms of m6A modification

According to the MODOMICS database, 163 different RNA posttranscriptional modifications had been identified by the end of 2017 [16]. More importantly, over 60% of all RNA modifications are methylation modifications, and m6A is a highly abundant and conserved mRNA modification in mammals. Previous experiments have shown that m6A modification mainly occurs in the adenine of the RRACH sequence (the m6A consensus motif), and the average mRNA contains approximately 3–5 m6A modifications [17, 18].

**Fig. 1:**
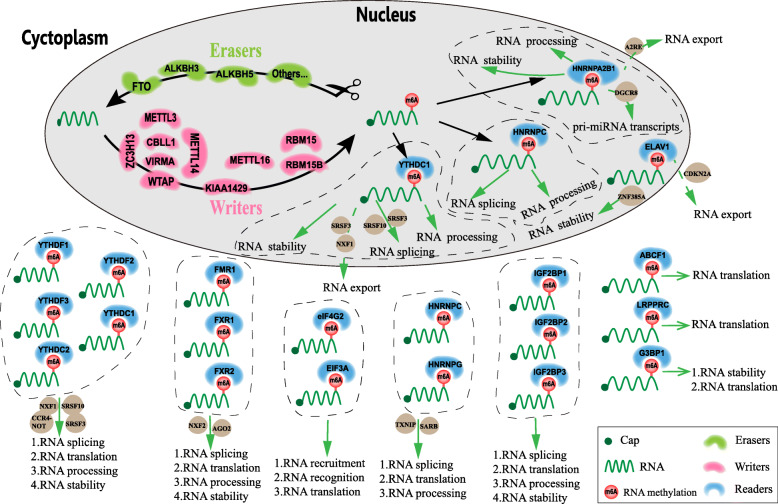
The mechanism of action of m6A modification in breast cancer. m6A modification consists of three vital components: writers, also termed methyltransferases; erasers, or demethylases, which remove the m6A modification; and readers, which recognize m6A-modified sites and further regulate m6A modification. m6A modifications affect RNA transcription, RNA processing, RNA splicing, RNA degradation, RNA export and RNA translation

In breast cancer, m6A modification regulates RNA termination codons, the 5′ cap structure, and the 3′ untranslated terminal region (UTR), and in this way affects RNA transcription, RNA processing, RNA splicing, RNA degradation and RNA translation (Fig. 1) [19, 20]. In general, m6A modifiers comprise three vital components: writers, also termed methyltransferases; erasers, or demethylases, which remove m6A modifications; and readers, which recognize m6A-modified sites and further regulate m6A modification [20, 21]. To date, known writers are METTL3, METTL14, METTL16, and WTAP. Known erasers include only demethylases, including FTO, ALKBH5 and ALKBH3. Readers mainly includemembers of the YTH family, HNRNP family, FXR family, IGF2BP family, eIF family, and G3BPs.

Previous studies have demonstrated that m6A modification indirectly/directly regulates target genes, thereby promoting or inhibiting tumour growth, proliferation, migration, invasion, and metastasis in breast cancers (Fig. 1, Table 1). Moreover, m6A modification patterns have therapeutic implications and correlate with drug resistance. In addition, m6A modification is associated with the clinicopathological features and prognosis of breast cancer (Table 2).

**Table 1:** Multiple functions exerted by RNA m6A methylation in various breast cancer cell lines

Types	Component	Regulation	Sources	Subtypes	Related targets	Key biological function	Ref.
Writers	MELLT3	Up-regulation	MCF-7 MDA-MB-231 MDA-MB-453 MDA-MB-468 MCF-10A	Luminal A Basal Her2+ Basal Basal	let-7 g, Bcl-2	1.METTL3 accelerate cell proliferation and migration. 2.Knockdown METTL3 reduce proliferation and accelerate cell apoptosis.	[22, 23]
		Down	MDA-MB-231 MDA-MB-468	Basal Basal	COL3A1	METTL3 overexpression suppress migration, invasion, and adhesion.	[24]
	RBM15	Uncertain	MCF-7, T47D	Luminal A	BAP1	RBM15 mediate cell growth and invasion.	[25]
	WTAP	Down	MCF-7 MDA-MB-231	Luminal A Basal	KGF, Erk1, Erk2	1.WTAP mediate proliferation and motility of breast cancer cells. 2.Prompt cell motility and invasion.	[26, 27]
	METTL14	Up-regulation	BT549 T47D, MCF-7 SKBR3 MDA-MB-231 MDA-MB-436	Basal Luminal A Her2+ Basal Basal	CXCR4, CYP1B1, Hsa-miR-146a-5p	1.LNC942 prompt cell proliferation and growth by targeting METTL14. 2.METTL14 effect hsa-miR-146a-5p expression and promote the migration and invasion, little effect on cell proliferation.	[28–30]
		Down	MDA-MB-231	Basal	–	Over expression of METTL14 remarkably suppressed cell growth and migration.	[26]
	KIAA1429	Up-regulation	MCF-7 SUM1315 MDA-MB-231	Luminal A Basal Basal	CDK1	KIAA1429 prompt proliferation, colony formation, migration, invasion, and metastasis.	[31, 32]
		Down	Breast cancer	Her2+	–	–	[26]
	CBLL1	Up-regulation	MCF-7 Hakai cells	Luminal A Luminal	ERα	CBLL1 inhibit cell proliferation and migration.	[33–35]
Erasers	FTO	Up-regulation	4 T1 MCF-7 SKBR-3 MDA-MB-231 MDA-MB-453	Basal Luminal A Her2+ Basal Her2+	BNIP, FTO/miR-181b-3p/ARL5B pathway	1.Promotes tumor growth and metastasis by inhibiting BNIP3. 2.Promote cell invasion and migration in vitro. 3.Promotes development and aggressiveness of breast cancer.	[36–40]
	ALKBH5	Up-regulation	MCF-7, T47D SUM-159 MDA-MB-231 MDA-MB-435	Luminal A Basal Basal HER2+	NANOG	1.HIF1α and HIF2α prompt ALKBH5expession. 2.ALKBH5 is required for breast cancer tumorigenicity and lung metastasis. 3.ALKBH5 knockdown to increased proliferation and migration.	[26, 30]
Readers	FMR1	Up-regulation	4 T1	Basal	E-cadherin, Vimentin	1.FMR1 prompt tumor growth, metastasis and cell invasion. 2.CHIP up regulates FMR1 expression.	[41–43]
	FXR1	Up-regulation	BT549 MCF-7 MDA-MB-231	Basal Luminal A Basal	ECT2, PRKCI, Myc	1.FXR1 prompt cell invasion.	[44–46]
	IGF2BP1	Up-regulation	T47D MDA-MB-231MDA-MB-157	Luminal A Basal Basal	IGF2, ACTB, MYC, CD44, CTNNB, BTRC, β-catenin	IGF2BP1 inhibit cell proliferation, tumor growth and pulmonary metastatic tumor.	[47–52]
	IGF2BP2	Up-regulation	SKBR3 MCF-7 MDA-MB-231MDA-MB-435MDA-MB-468 LM2-4 cells	Her2+ Luminal A Basal Basal Basal Basal	CTGF, IGF-2, IGF-1, ERK, PI3K/Akt pathways	1.IGF2BP2 prompt cell migration and reduced cell adhesion by targeting CTGF mRNA. 2.IGF2BP2 stimulate cell proliferation, growth and differentiation. 3.miR-1193 also suppressed IGF2BP2 translation. 4.IGFBP2 mediate endothelial recruitment through IGF1/IGF1R pathways.	[53–56]
	IGF2BP2/IGF2BP3	Up-regulation	MDA-MB-157 MDA-MB-231 MCF-7, T47D BT474	Basal Basal Luminal A Luminal B	microRNA-200a, CNOT1	IGF2BP2/IGF2BP3 cooperate increase cell migration and invasion by targeting microRNA-200a.	[57]
	IGF2BP3	Up-regulation	T47D, MCF-7 HCC1937 SUM-1315 MDA-MB-231MDA-MB-435 MDA-MB-468 SKBR3	Luminal A Basal Basal Basal Basal Basal Her2+	EMT, ABCG, EGFR signaling, CD44/CD44 + Fbs/IGF2	1.IGF2BP3 increase cell migration and invasion. 2.IGF2BP3 prompt EMT and Blockade of miR-3614 maturation.	[25, 57–67]
	HNRNPC	Up-regulation	T47D MCF-7 MDA-MB-435	Luminal A Luminal A Basal	IRF3/7, ISGF3, IFNβ	1.HNRNPC prompt cell proliferation, migration, invasion and metastasis. 2.Repression of HNRNPC arrested the proliferation and tumorigenesis.	[68–70]
	HNRNPA2B1	Up-regulation	MCF-7 MCF-10A MDA-MB-231 LCC9	Luminal A Basal Basal Luminal	STAT3, ERK1, ERK2	1.HNRNPA2B1 prompt in cancer development, progression, gene expression. 2.HNRNPA2B1 associate with endocrine resistance. 3.Knockdown of HNRNPA2B1 inhibit proliferation.	[71, 72]
		Down	MDA-MB-231 MCF-7 cells	Basal Luminal A	PFN2, Wnt pathway, ERK/MAPK/Twist pathway	HNRNPA2B1 inhibits the growth of xenograft tumours but promotes spontaneous lung metastasis.	[73]
	YTHDF1 YTHDF2	Up-regulation	MCF-7 MDA-MB-231 MDA-MB-468	Luminal A Basal Basal	–	1.YTHDF1/2 regulate the expression of YTHDF3. 2.YTHDF1/2 enhance cell proliferation, migration and invasion.	[17, 31, 36, 74–76]
	YTHDF3	Up-regulation	MDA-MB-231 HCC1954 4 T1	Basal Her2+ Basal	ST6GALNAC5, GJA1, EGFR	1.YTHDF3 promotes brain endothelial adhesion, extravasation, invasion, angiogenesis, and cell-astrocyte interaction. 2.Knockdown of YTHDF3 significantly decrease brain metastasis in mouse models. 3.miR-106b-5p down regulate the expression of YTHDF3.	[68, 77]
	eIF3A	Uncertain	MCF-7 ZR-75-1 MDA-MB-231 MDA-MB-453MDA-MB-468	Luminal A Luminal B Basal Her2+ Basal	4EBP1, M2 protein	eIF3A facilitate the proliferation or migration.	[78–81]
	G3BP1	Up-regulation	MCF-7 SKBR3 MDA-MB-231 MDA-MB-468 BT549	Luminal A Her2+ Basal Basal Basal	PMP22, EMT, ZEB1, TGF-b signaling	1.G3BP1 facilitate tumor invasion and migration. 2.G3BP1 involve in vesicle trafficking.	[82–86]

**Table 2:** The relationship between RNA m6A methylation and Clinical features in breast cancer patient

Types	Component	Regulation	Sources	Clinicopathological features and prognosis	Ref.
Writers	MELLT3	Up-regulation	Normal and Luminal subtypes	MELLT3 associate with worse prognosis.	[23]
		Down	TNBC	MELLT3 associate with better DMFS and OS.	[24, 26]
	WTAP	Down	Breast	Reduced of METTL14 have poor DMFS.	[26]
	METTL14	Up-regulation	Normal, Luminal subtypes	1.Reduced of METTL14 have poor DMFS. 2.METTL14 associate with T staging, without molecular Typing, microvascular invasion, nerve invasion and metastasis.	[26]
	KIAA1429	Down	HER2+ subtype	KIAA1429 associate with intrinsic subclasses, nodal metastasis, and worse OS.	[32]
	CBLL1	Up-regulation	Breast cancer	CBLL1 associate with ER status.	[34]
	ZC3H13	Down	TNBC	Have no association with OS	[87]
Erasers	FTO	Up-regulation	HER2+, Normal, Luminal subtypes, Invasive ductal carcinoma.	1.FTO associate with advanced progression, peritumoral lymphovascular invasion, lymph node metastasis, TNM stage, HER2 status and ER/PR status. 2.FTO associate with shorter DFS/OS/RFS.	[37, 88, 89]
	ALKBH5	Up-regulation	Breast cancer	ALKBH5 have no association with DMFS.	[26]
Readers	YTHDF1 YTHDF2 YTHDF3	Up-regulation	Breast cancer	1.YTHDF1 associate worse OS and RFS 2.YTHDF2 associate brain metastasis 3.YTHDF3 associate with worse OS and RFS	[31, 68, 74]
	FMR1	Up-regulation	Breast cancer	FMR1 associate with high tumor grade (G3) and high Ki67 and easier metastasis to the lungs	[42]
	FXR1	Up-regulation	TNBC	FXR1 associate with worse pCR and poor DFMS and OS.	[46, 58]
	HNRNPA2B1	Up-regulation	Breast cancer	1.Have correlation with endocrine resistance. 2.Associated with worse prognosis	[41]
		Down	Breast Cancer	Associated with better OS.	[90]
	IGF2BP1	Up-regulation	Breast cancer	IGF2BP1 does not affect tumor growth or size.	[55]
	IGF2BP2	Up-regulation	Breast cancer	IGF2BP2 correlate with advanced stage and poor survival.	[55, 91]
	IGF2BP3	Up-regulation	TNBC	1.IGF2BP3 associate with larger size, higher grade, higher clinical stage, necrosis, CK5/6. 2. IGF2BP with worse DFS/OS. 3.IGF2BP3 promote chemoresistance to doxorubicin and mitoxantrone.	[62, 63]
	G3BP1	Up-regulation	Breast cancer	G3BP1 associate with tumor response to the Akt-inhibitor MK-2206.	[82]

*Abbreviations*: *TNBC* triple negative breast cancer, *RFS* relapse free survival, *DFS* disease free survival, *OS* overall survival, *DMFS* distant metastasis free survival

### M6A modification modulates biological processes in breast cancer

#### Methyltransferases/writers in breast cancer

Methyltransferase-like 3 (METTL3) and methyltransferase-like 14 (METTL14) form a stable methyltransferase complex and play a key role in localization recognition [92–94]. MELLT3 acts as a catalyst in the cell nucleus and rapidly initiates the formation of the methyltransferase complex. MELLT14 prompts complex binding to relevant RNA sites and recognizes the substrate. In addition, METTL14 induces serine phosphorylation on METTL3.

Previous studies indicated that METTL3 is significantly highly expressed in breast tissues and cells [14, 17, 22, 23, 26, 95]. Hong Wang et al. showed that silencing METTL3 reduces methylation levels, represses proliferation and induces apoptosis by targeting Bcl-2 [23]. Hepatitis B X-interacting protein (HBXIP) upregulates METTL3 expression and then accelerates the proliferation of breast cancer cells by inhibiting the tumour suppressor let-7 g [22]. Similarly, silencing METTL3 inhibits cell growth and proliferation and accelerates cell apoptosis [22]. LINC00942 (LNC942), an oncogene, promotes METTL14 expression and induces cell proliferation and growth by targeting CXCR4 and CYP1B1 [28]. In addition, METTL14 affects hsa-miR-146a-5p expression and promotes migration and invasion but has little effect on cell proliferation [29].

In contrast to previous studies, Wu et al. revealed that METTL3 and METTL14 expressions are lower in breast tissues than in normal tissues [26]. Interestingly, however, the expressions of METTL3 and METTL14 were still higher in normal and luminal types of breast cancer. In addition, overexpression of METTL14 and/or knockdown of ALKBH5 remarkably suppressed cell migratory abilities [26]. A recent study of triple-negative breast cancer (TNBC) also found that METTL3 expression is lower in breast cancer cells. METTL3 inhibits the metastasis of TNBC cells by downregulating the expression of COL3A1 [24]. Nate J. Fry et al. demonstrated that tumour cell proliferation and migration are stimulated by upregulating the expression of METTL3 and METTL14 or downregulating the expression of ALKBH5 [30]. Another study indicated that the expression of METTL14 and METTL16 was not significantly enhanced in MDA-MB-231, MDA-MB-468 and MCF-7 cell lines [31].

Wilms tumour 1-associating protein (WTAP) regulates G2/M cell cycle transition by binding to the 3′ UTR of CCNA2 and promotes cell growth and migration by combining with METTL3/METTL14 [26]. Keratinocyte growth factor enhances the expression of WTAP and thereby increases the proliferation of breast cells [27]. However, another study showed that WTAP expression was not significantly different in 20 breast cancer tissue samples compared with paired normal tissues [26].

KIAA1429, also called VIRMA, is mainly expressed in the cytoplasm in most human breast cancer cell lines and regulates m6A methylation modification as an RNA binding protein. Studies have shown that KIAA1429 is highly expressed in breast cancer tissues and expressed at low levels in pericarcinous tissues [31, 32]. In the MCF-7, MDAMB-231 and SUM1315 cell lines, KIAA1429 facilitates cell proliferation, colony formation, migration, invasion, and metastasis by regulating the expression of CDK1 [32]. KIAA1429 overexpression predicts poor overall survival in breast cancer patients [31]. Another study showed that KIAA1429 is expressed at lower levels in HER2-positive breast cancer [26].

RBM15/RBM15B, RNA binding motif protein 15/15B, promotes the methylation of RNAs by binding to uracil-enriched regions [25]. RBM15, as an oestrogen-responsive gene, regulates cell growth in MCF7 and T47D cell lines [25]. Further research found that BARX2 and oestrogen receptor-a (ESR1) co-ordinately modulate cell growth and cell invasion by affecting the expression of RBM15 [25]. It is well known that RBM15 impacts diseases, and how RBM15 is regulated is an important question; thus far, nothing is known about this issue. RBM15B, located in close proximity to the 3p21 tumour suppressor region, is highly positively correlated with BRCA1-associated protein-1 expression in both black and white female patients with invasive breast carcinoma [96].

Eun-Yeung Gong et al. showed that CBLL1, also named HAIKI or RNF188, is mainly expressed in the cytoplasm in most human breast cancer cell lines [33]. CBLL1, as an E3 ubiquitin protein ligase, competes with ERa coactivators and plays a negative role in the development and progression of breast cancers [34]. A study published in 2009 that enrolled 22 samples found that CBLL1 expression has no correlation with invasive ductal breast carcinomas and normal adjacent tissue [35]. However, CBLL1 mRNA expression was higher (samples 83.3% ER positive) in these breast cancers than in adjacent tissues.

#### Demethylases/erasers in breast cancer

FTO, as a member of the AlkB family, is the first identified m6A demethylase/eraser, which maps to chromosome 16q12.2 and is highly expressed in most breast cancers [36–38, 88, 89]. For MDA-MB-231, MCF-7 and 4 T1 breast cell lines, upregulated FTO promotes tumour proliferation and metastasis or reduces cell apoptosis by targeting BNIP3. Furthermore, the article highlighted that silencing FTO can inhibit the growth of breast tumours in vivo [88]. In HER2-positive breast cancer, FTO also reinforces cell invasion and migration in vitro through the FTO/miR-181b-3p/ARL5B signalling pathway [89]. Recently, one study also illustrated that FTO expression may play an essential role in the development and aggressiveness of breast cancer, especially HER2-overexpressing breast cancer [37]. One researcher showed that FTO expression is low in HER2-positive breast cancer [26].

ALKBH5, another member of the AlkB family, is highly expressed in most breast cancers [26]. ALKBH5 may function as an m6A reader in the cytoplasm and cytosol and promote the translation of its target mRNA [14, 95]. Recently, it was reported that ALKBH5 functions as a tumour promoter in the pathogenesis of breast cancer [39, 95]. ALKBH5 plays a crucial role in tumorigenicity and lung metastasis. Furthermore, under anaerobic conditions, HIF1α and HIF2α significantly increase ALKBH5 mRNA expression [40, 97]. Moreover, knockdown of ALKBH5 expression significantly inhibits tumour formation and decreases breast cancer stem cell number in breast tumours [40]. In addition, ALKBH5, as an oncogene, regulates the self-renewal and proliferation of breast cancer tumour stem cells.

#### Readers in breast cancer

##### The YTH family

Previous studies found that members of the YTH family play a crucial role in RNA stability, RNA splicing, RNA translation, and RNA processing in cancers [17, 31, 74, 77, 88, 98, 99]. The YTH family mainly includes the following members: YTHDF1, YTHDF2, YTHDF3, YTHDC1, and YTHDC2. YTHDF2 is the first reader to be associated with m6A modification and mediate mRNA processing [74, 77]. In the cytosol, YTHDF2 regulates mRNA stability and degradation via interaction with CCR4-NOT.

YTHDF1 and YTHDF3 can promote RNA translation in HeLa cells. In the nucleus, YTHDC1 regulates mRNA splicing, mRNA export, RNA degradation and partly RNA transcripts by recruiting certain binding sites [17, 31, 74, 77, 88, 99]. In breast cell lines, YTHDF1 and YTHDF2 are mainly located in the cytoplasm, while YTHDC1 is mainly located in the nucleus [31]. In addition, YTHDF1, YTHDF2 and YTHDF3 are highly expressed in breast cancer and enhance cell proliferation, migration and invasion [31, 75, 98]. Notably, YTHDF3 expression was higher in brain metastases than in parental cells [75]. Furthermore, knockdown of YTHDF3 significantly decreased brain metastasis in mouse models. YTHDF3 copy number gain is higher in breast cancer with brain metastases than in primary breast tumours. Interestingly, in a separate study, miR-106b-5p gene transfection downregulated the expression of YTHDF3, which enhanced cell proliferation, migration and invasion of oestrogen-induced breast cancer [99]. Furthermore, in cells transfected with mimics, researchers detected that miR-106b-5p regulates YTHDF3 expression by suppressing translation or inducing mRNA degradation.

##### The HNRNP family

HNRNP family members, including HNRNPC, HNRNPG and HNRNPA2B1, also modulate RNA stability, RNA splicing, RNA translation, and RNA processing. HNRNPC, a splicing factor, is highly expressed in breast cancer and promotes tumour cell proliferation and growth [68, 76]. Moreover, HNRNPC is involved in aberrant splicing and implicated in the formation of the tumour transcriptome [69]. In MCF7 and T47D, but not MCF10A, BT549, and MDA-MB-231 cells, even partial suppression of HNRNPC function can result in a type I interferon response, an increase in endogenous dsRNA, and upregulation of ISG expression via activation of the ISGF3 complex. Knockdown of HNRNPC inhibits cell proliferation and tumour growth of these breast cancer cells [68]. Another study found that HNRNPC exhibits considerably higher expression in LvBr2 than MDA-MB-435 cells [68, 70]. In LvBr2 cells, miR-146a inhibits the activation of Akt by downregulating HNRNPC and thereby suppresses tumour migration and invasion.

HNRNPA2B1, another splicing factor of breast cancer, is also significantly upregulated in human breast cancer tissues and cell lines [71]. Emerging evidence has shown that HNRNPC plays a major role in cancer development, progression, gene expression, and signal transduction. Knockdown of HNRNPA2B1 inhibits cell proliferation and cell growth in both the MCF-7 and MDA-MB-231 cell lines. In addition, in MCF-7/H1 and 231/H2 cells with HNRNPA2B1 knockdown, the levels of phospho-STAT3 and phospho-ERK1/2 expression were significantly decreased. That is, HNRNPA2B1 regulates the STAT3 and ERK1/2 signalling pathways in breast cancers. Notably, HNRNPA2B1 expression is higher in LCC9 tamoxifen-resistant cells than in parental tamoxifen-sensitive MCF-7 cells [72]. Conversely, HNRNPA2B1 expression is significantly decreased in invasive breast cancer (GSE59246) and MDAMB-231 and MCF-7 cells [73]. HNRNPA2B1 inhibits the growth of xenograft tumours but promotes spontaneous lung metastasis. HNRNPA2B1 knockdown promotes F-actin cytoskeleton formation, activates the downstream genes of the Wnt pathway, activates the ERK/MAPK/Twist pathway, upregulates PFN2 expression, and inhibits the epithelial-mesenchymal transition and metastasis (EMT)-promoting genes and signalling pathways [73]. HNRNPG, as a specific regulator of ERa exon 7 splicing, regulates oestrogen receptor alpha expression in endometrial cancer [100]. However, little is understood about the molecular characteristics of HNRNPG in breast cancer. Based on these findings, HNRNPG may play a potential role in breast cancer with oestrogen receptor expression.

##### The FXR family

Three members of the FXR family, FMR1, FXR1 and FXR2, participate in Fragile X Mental Retardation Syndrome by forming multiprotein complexes [41, 90]. Previous research found that all FXR family proteins contain two KH domains and one RCG box and associate with polyribosomes, predominantly with 60S large ribosomal subunits [90].

FMR1, containing a CGG trinucleotide repeat element in its 5′ untranslated region, is highly expressed in human breast cancer and distal metastasis and affects cell-cell adhesion, cell shape and invasion of 4 T1 cell lines [41, 42]. FMR1 also mediates E-cadherin and Vimentin expression. Meanwhile, CHIP interacts with FMR1 via a nontetratricopeptide repeat domain and upregulates FMR1 expression [43].

FXR1, as an RNA-binding protein, plays a vital role in breast cancer progression and cell invasion [44, 45]. In TNBC, FXR1, as a novel prognostic biomarker, stimulates lung metastasis FXR1 was associated with better pCR to neoadjuvant chemotherapy in TNBC [44]. FXR1, FXR2 and hnRNPK are required for these treRNA functions, while their expression promotes tumour invasion in vitro and metastasis in vivo [46]. FXR1 or FXR2 did not mediate cell proliferation in MCF7 cells. Moreover, knockdown of hnRNPK or FXR1 but not FXR2 increased the tumour growth of mouse model with MCF7 cells, whereas double knockdown or triple knockdown of the RNA-binding proteins significantly decreased primary tumour growth. That is, FXR1 mediated tumour growth not cell proliferation. Briefly, the combination of hnRNPK and FXR2 affects tumour metastasis in vivo, however, the curative mechanism need to be evaluated.

##### The IGF2BP family (KH domain proteins)

Insulin-like growth factor 2 mRNA binding proteins (IGF2BPs) recruit target transcripts to cytoplasmic protein-RNA complexes [47, 53, 54, 58–60, 101–103]. IGF2BP1, IGF2BP2 and IGF2BP3, members of the IGF2BP family, modulate RNA splicing, translation, processing, and stability and thereby affect cell proliferation, differentiation, invasion and metastasis in breast cancer. IGF2BPs, as blinding proteins of FGF13-AS1, prevent targeted Myc mRNA degradation to regulate breast cancer cell proliferation, invasion and metastasis [102].

IGF2BP1, as a binding protein, regulates cell apoptosis by inhibiting the accumulation of eIF5A [104]. Previous studies found that IGF2BP1 is highly expressed in breast cancer [47–50]. IGF2BP1 promotes the stability of β-catenin mRNA via the β-catenin/TCF4 response element and then accelerates cell adhesion and transcription of breast tumours [47]. Research in mouse xenograft models demonstrated that IGF2BP1 inhibits cell proliferation, tumour growth and tumour metastasis of breast cancer by binding to the 3′UTR of GDF15 mRNA [49, 50], while other research showed that IGF2BP1 does not affect tumour growth or size [51]. In T47D and MDA231 cells, IGF2BP1 plays a crucial role in repressing cell migration and metastasis, partly through the regulation of E-cadherin mRNA expression [50]. In T47D cells, silencing IGF2BP1 resulted in increased levels of UCA1 and thereafter increased the expression of miR-122-5p target mRNAs, eventually prompting breast cancer cell invasion [52].

IGF2BP2, as an oncofoetal protein, is located on chromosome 3q27 and is highly overexpressed in breast cancer cells and tissues [53, 55, 56]. Previous studies demonstrated that knockdown of IGF2BP2 significantly reduced metastatic endothelial density and functional vessel content and thus suppressed lung metastasis of breast tumour cells. IGF2BP2 overexpression increases cell migration and reduces cell adhesion by targeting CTGF mRNA in vitro and stimulates cell proliferation and influences growth and differentiation [56]. Additionally, in MDA-MB-231 and MCF-7 cells, miR-1193 suppresses the expression and activation of the ERK and PI3K/Akt signalling pathways by inhibiting IGF2BP2 translation and reducing the proliferation and invasion of human breast cancer cells [54]. CCN6 directly reduces IGF2BP2 and HMGA2 expressions and further reduces the tumour growth of MDA-MB-231 cells [53].

Emerging evidence has shown that IGF2BP3 is highly expressed in breast cancer [25, 57–67]. Interestingly, IGF2BP3 expression is higher in mesenchymal cells than in epithelial cells [105]. Hye-Youn Kim et al. elucidated that IGF2BP2 and IGF2BP3 cooperate to provoke cell migration, invasion, and invasion by negatively targeting microRNA-200a and partially determining the characteristic phenotype [61]. In breast cancer cells, IGF2BP3 has a direct correlation with E-cadherin, vimentin, and Slug (*P* < 0.05) and further promotes invasion and migration by prompting the EMT^83^ and reducing cell apoptosis by activating the Hedgehog signalling pathway CD44/CD44^+^Fbs/IGF2 [59, 106]. Moreover, IGF2BP3 mediates cell migration, invasion and stem cell properties through EGFR signalling and repression by ERβ, SLUG mRNA, and miRNA-34a [105]. IGF2BP3 increases TRIM25 expression by inhibiting miRNA-3614 maturation, thereby prompting cell growth and proliferation [58]. IGF2BP3, as an RNA-binding protein for CERS6-AS1, prompts CERS6 mRNA stability and then accelerates cell proliferation and suppresses cell apoptosis [60].

##### The eIF family with C-terminal readers

EIF3A, an oncogene in human breast cancer, was first described as the largest subunit of the eIF family by Bachmann et al. [78]. It has been reported that eIF3A is significantly highly expressed in breast cancer [79–81]. Dong et al. found that eIF3A regulates the synthesis of M2 protein, and decreasing eIF3A expression in MCF7 cells significantly reversed their malignant growth phenotype [107]. TG2^/TIS21^ inhibited translational initiation by depleting eIF availability by inhibiting 4EBP1 phosphorylation [80]. In addition, rs10787899 and rs3824830 SNPs in eIF3A were associated with an increased risk of breast cancer (all *P* < 0.01) [81]. EIF3A, also described as an oestrogen-responsive gene, can target ER-mediated signals and facilitate the cell proliferation and/or migration of ERα-positive breast cancer cells [79]. More experiments are needed to identify whether downregulation of eIF3A expression can change the malignant phenotype of breast cells.

In MCF7 cells, suppressing eIF4G1 facilitates the expression of eIF4G2, while suppressing eIF4G2 partially increases sensitivity to ionizing radiation-mediated DNA damage [108]. Columba et al. revealed that eIF4G1 has a very weak interaction with DAP5 [109]. Additionally, ERα − breast cancer patients have higher expression of eIF4G2 than ERα + patients. Moreover, eIF4G2 is abundantly expressed in proliferating cells, and the downregulation of eIF4G2 levels decreases the rate of global protein translation and inhibits cell proliferation [110].

##### Other readers in breast cancer

G3BP1 is dramatically overexpressed in breast cancer [82–86]. G3BP1 promotes cell proliferation by inhibiting PMP22 mRNA expression in breast cells and facilitates tumour metastasis and invasion by inhibiting the expression of E-cadherin and by increasing the expression of TGF-β signalling genes, Smad target genes, vimentin, Snail, Slug, fibronectin and ZEB1 [83, 84, 111]. Moreover, overexpression of G3BP1 induces the EMT in MCF-7 cells, thereby increasing cell migration and invasion [86]. In addition, downregulated G3BP1 restricts the invasion and migration of MDA-MB-231 cells compared to upregulated G3BP1, facilitating the tumour invasion and migration of MCF-7 cells [83, 84]. In addition, G3BP1 inhibits cell apoptosis by restraining nuclear p53 levels [112]. More interestingly, G3BP1 is also involved in vesicle trafficking [113].

### The relationship between m6A modification and clinical features

#### Writers in breast cancer

Recent research by Hong Wang et al. found that high METTL3 expression was associated with worse prognosis in breast cancer (*P* = 0.002) [23]. Patients with METTL3, METTL14, WTAP and FTO overexpression have better metastasis relapse-free survival [26]. METTL3 overexpression was associated with better distant metastasis-free survival (*P* = 0.023) and extended OS (*P* = 0.042) [24]. In addition, METTL14 is associated with T staging, without molecular typing, microvascular invasion, nerve invasion and metastasis [26]. The 5-year overall survival of patients with KIAA1429 and CDK1 overexpression was significantly lower than that of patients with low expression of KIAA1429, CDK1 or both (*P* = 0.021) [32].

One study also found that surviving patients, regardless of race, have a higher level of RBM15B expression than those who do not survive [96]. That is, RBM15B may suppress tumour growth in these breast patients [96]. ZC3H13 had no association with the OS of TNBC from TCGA downloaded dates (*n* = 115, *P* = 0.9623) [87].

#### Erasers in breast cancer

FTO upregulation is significantly associated with shorter overall survival in patients with advanced-stage breast cancer [88]. Additionally, FTO overexpression correlates with tumour size, nuclear grade, peritumoral lymphovascular invasion, lymph node metastasis, and TNM stage. FTO overexpression implies poor DFS/OS/RFS for HER2-positive breast cancer [89]. In breast cancer, the percentage of FTO-positive expression in the HER2 overexpression subtype (97.1%) was significantly higher than that in the triple-negative (76.2%) and luminal subtypes (52.2%) (*P* < 0.001) [37]. Moreover, FTO expression has a positive correlation with HER2 status and ER/PR status. However, FTO has no association with age, tumour size, lymph node status, TNM stage, histological grade, Ki67, or BMI in breast cancer [37]. Additionally, ALKBH5 expression has no association with metastasis relapse-free survival [26].

#### Readers in breast cancer

Both YTHDF1 and YTHDF3 overexpression correlated with poor overall survival in breast cancer patients (all *P* values < 0.05) [31, 98]. YTHDF3 overexpression is correlated with lower relapse-free survival rates [31]. In the BCIP breast cancer database, patients with high HNRNPA2B1 expression (*n* = 30) had longer survival times than patients with low HNRNPA2B1 expression (*n* = 288) [73]. In contrast, breast cancer patients with high HNRNPA2B1 expression have worse survival [72].

FMR1 correlates with HER2 status, ER status, higher tumour grade (G3) and higher Ki67 expression. FMR1 overexpression correlated with an increased probability of breast cancer progression, particularly metastasis to the lungs [41]. FXR1 overexpression is associated with worse disease-specific disease-free survival in the METABRIC breast cancer cohort and worse overall survival in the TCGA cohort [45]. FXR1 overexpression is associated with worse distant metastasis-free survival in TNBC patients (*n* = 16, *P* = 0.01, HR = 9.63, 95% CI = 1.7–43.96) but not in TNBC patients [44].

Research in mouse xenograft models demonstrated that IGF2BP1 inhibits pulmonary metastatic tumours of breast cancer [49, 50], while other research showed that IGF2BP1 does not affect tumour growth or size [51]. In patients with early-stage breast cancer, IGF2BP2 overexpression is also correlated with short survival, and therefore IGF2BP2 may be a useful serum biomarker for breast cancer screening and diagnosis [55, 91]. From one cohort of 96 breast cancer patients, IGF2BP2 expression was significantly increased in patients with stages III and IV versus stages I and II. In TNBC, IGF2BP3 is associated with a larger tumour size, higher grade, higher clinical stage, necrosis, and CK5/6 expression [63]. In metaplastic breast carcinoma and TNBC, IGF2BP3 is associated with reduced DFS and OS in patients [62, 63]. Based on previous findings, eIF3A can be a tumour marker of invasive ductal breast carcinomas, but its expression had no association with age, tumour size, differentiation grade, nodal status, or DNA index of breast cancer patients [78].

### The role of m6A in lncRNA regulation and cancer stem cells in breast cancer

Emerging evidence demonstrates that long noncoding RNAs (lncRNAs) and cancer stem cells play critical roles in human breast cancer (BC) by interacting with m6A. A previous study indicated that lncRNA KB-1980E6.3 recruited IGF2BP1 to maintain breast cancer stem cell stemness by targeting c-Myc [114]. In breast cancer, METTL3-induced LINC00958 upregulation affects tumorigenesis via the miR-378a-3p/YY1 axis [115]. In addition, ALKBH5 overexpression decreased the percentage of breast cancer stem cells [40]. In MDA-MB-231 cells, the knockdown of ALKBH5 significantly reduced the number of breast cancer stem cells [40].

### Impact of m6A modification on therapy resistance

Moreover, 5′-fluorouracil (5-FU), but not doxorubicin, paclitaxel, and cyclophosphamide, reduces the expression of KIAA1429 in breast cancer cells [32]. YTHDF1 also has a positive correlation with molecular subtypes and nodal metastasis [98]. In MCF-7 cells, HNRNPA2B1 overexpression reduces sensitivity to 4-hydroxytamoxifen and fulvestrant [72]. IGF2BP2 could be a pathway and specific activity of MEK1/2 [101]. In TNBC cells, IGF2BP3 promotes chemoresistance to doxorubicin and mitoxantrone by regulating breast cancer resistance protein (ABCG2) [64]. In addition, a previous study identified that G3BP1 is associated with the tumour response to the Akt inhibitor MK-2206 [82].

## Discussion and future perspectives

This article describes the role, mechanism and clinical application of m6A in breast cancer. RNA m6A modification plays an important role in promoting or inhibiting tumour growth, proliferation, migration, invasion, specific metastasis, drug resistance and prognosis of breast cancer through three effectors: writers, erasers and readers.

For breast cancer, different researchers have presented inconsistent results on the expression levels of m6A-related proteins, including MELLT3, MELLT14, KIAA1429, and HNRNPA2B1. MELLT3 and MELLT14 were upregulated in breast cancer [23] and downregulated in TNBC [24]. KIAA1429 was upregulated in luminal A and basal breast cancer and downregulated in Her2+ breast cancer. The expression of HNRNPA2B1 was decreased in invasive breast cancers in the GEO database (GSE59246) [73] and was increased in LCC9 breast cancer cells and in Asian patients [71, 72]. These differences have been explored by previous researchers, and there are several reasons that might account for the inconsistent results. First, the enrolled patients had different molecular typing. Second, these studies included patients of different races.

In addition, although m6A modification showed biological effects against breast cancers, there are still some questions that require further investigation and clarification. First, m6A modification has potential as a biomarker in early diagnosis. In patients with early-stage breast cancer, IGF2BP2 may be a useful serum biomarker for breast cancer screening and diagnosis [55, 91]. However, the application of this marker is often restricted by difficulty in obtaining sufficient samples for testing. Thus, large-scale clinical trials on m6A modification are necessary. Second, until now, most studies on m6A modification have tended to focus on its mechanism in breast cancer cell lines. Therefore, more conversion studies are needed to clarify whether m6A alone or in combination with other therapies could be used to treat breast cancers, even other solid tumours. Third, SNPs in eIF3A were associated with an increased risk of breast cancer [81]. It is also necessary to further speculate the impact how the mutations mediate m6A modification. Furthermore, m6A modifications can be targeted by specific drugs to improve drug resistance to breast cancer. Finally, the interactions between m6A and gene mutation frequency, genome copy number variation, and specific molecular subtypes remain unclear and need to be explored.

## Conclusion

Recently, with the rapid development of bioinformatics and translational medicine methods and improvements in m6A editing tools and RNA sequencing, it is hopeful that RNA m6A modification can be developed as tumour markers for diagnosis and as drug targets for breast cancer treatment. There are three major types of m6A modification effectors, namely, writers, erasers and readers, in the human body. Thus, m6A modification is also likely to become a new multitargeting inhibitor that is efficacious against breast cancer.

## Data Availability

Not applicable.

## Abbreviations

m6A: N6-methyladenosine
TNBC: Triple-negative breast cancer
METTL3: Methyltransferase-like 3
METTL14: Methyltransferase-like 14
RFS: Relapse-free survival
DFS: Disease-free survival
OS: Overall survival
DMFS: Distant metastasis-free survival
